# Increases in Circulating Cortisol during the COVID-19 Pandemic are Associated with Changes in Perceived Positive and Negative Affect among Adolescents

**DOI:** 10.1007/s10802-022-00967-5

**Published:** 2022-09-01

**Authors:** Brittany K. Taylor, Madison H. Fung, Michaela R. Frenzel, Hallie J. Johnson, Madelyn P. Willett, Amy S. Badura-Brack, Stuart F. White, Tony W. Wilson

**Affiliations:** 1 Institute for Human Neuroscience, Boys Town National Research Hospital, 378 Bucher Circle, Boys Town, Omaha, NE 68010 USA; 2 Department of Pharmacology and Neuroscience, Creighton University, Omaha, NE USA; 3 Department of Psychological Science, Creighton University, Omaha, NE USA

**Keywords:** Hair cortisol concentration (HCC), Mental health, Youth, Coronavirus, Longitudinal, Stress

## Abstract

The Coronavirus Disease 2019 (COVID-19) pandemic has spread across the world and resulted in over 5 million deaths to date, as well as countless lockdowns, disruptions to daily life, and extended period of social distancing and isolation. The impacts on youth in particular are astounding, with shifts in learning platforms, limited social outlets, and prolonged uncertainty about the future. Surveys have shown that mental health among youth has severely suffered during the pandemic. However, limited research to date has reported on physiological indices of stress surrounding the pandemic, such as cortisol. Cortisol is a stress hormone that typically increases during stressful situations and can have deleterious effects on mental and physical health when chronically heightened. The present study leveraged hair cortisol concentration measurements, which allowed the retrospectiveinvestigation of circulating cortisol prior to- versus after pandemic-related local lockdowns during the first wave of the pandemic. A final sample of 44 youth ages 10- to 18-years-old provided hair samples and reported on their perceived affective well-being and level of concern regarding pandemic-related stressors between May and June of 2020. We found significant levels of concern and decreases in affective well-being following local lockdowns. Moreover, we saw that cortisol robustly increased following local lockdowns, and those increases were predictive of changes in affect. These findings provide critical insights into the underlying neuroendocrinology of stress during the pandemic and support the need for resources to support youths’ mental health and well-being during this globally significant event.

**Supplementary Information:**

The online version contains supplementary material available at 10.1007/s10802-022-00967-5.

## Introduction

Catastrophic global events can have reverberating consequences on mental health for extended periods. Major events like natural disasters and war are known to have both acute and chronic impacts on physiological stress responses, as well as perceived psychological distress and mental health (Miller et al., 2007; Stalder et al., 2017). For over a year now, the Coronavirus Disease 2019 (COVID-19) pandemic has spread across the world, killing over 5 million people, damaging economies, overburdening healthcare systems, and physically and psychologically harming individuals (Courtney et al., 2020; Taylor et al., 2021). The United States was hit particularly hard by the pandemic early on, harboring nearly 20% confirmed cases and 15% of global deaths (https://coronavirus.jhu.edu/). The gravity of the situation during the first wave forced intermittent, sometimes prolonged school closures across the country (Auger et al., 2020), leaving youth disconnected from critical resources (Fisher et al., 2020; Viner et al., 2020).

Notably, a rapidly growing body of research consistently shows that during the pandemic, youth are reporting decreased social support (Poletti & Raballo, 2020), challenges with shifting learning platforms (Petretto et al., 2020), and generalized decreases in mental health (Cost et al., 2021; Courtney et al., 2020; Glynn et al., 2021; Liang et al., 2020; Racine et al., 2021). Multiple reports suggest that globally, lockdowns and the associated outcomes (e.g., reduced access to services, social isolation) are associated with increased depression, anxiety, irritability, and even alcohol and substance use among adolescents (Newlove-Delgado et al., 2021; Panchal et al., 2021; UNICEF Innocenti Research Centre, 2021). This is crucial given that mental health disorders often develop during adolescence (Bitsko et al., 2018; Merikangas et al., 2010), and given that early stressful and traumatic experiences are known to have prolonged consequences for physical and mental health across the lifespan (Conway et al., 2016; Miller et al., 2011).

Studies examining the impact of the pandemic on mental health are critical to informing public health responses and plans to address the mental health needs of youth. However, most studies reporting effects of the pandemic are based on ad hoc surveys distributed after local lockdowns were in place; there is limited longitudinal data from which researchers can determine shifts from baseline in response to the events of this global crisis. One such study explored self-reported psychological wellness among youths prior to and 2 months after lockdowns began, and found generally poorer outcomes during the pandemic that were more severe among female youths who felt socially disconnected from their peer groups (Magson et al., 2021). Another recent study leveraged a longitudinal population-based data set tracking self-reported mental health outcomes among youths in England in 2017 and again in 2020 shortly after the first wave of COVID-19 lockdowns (Newlove-Delgado et al., 2021; Vizard et al., 2020). The study found a significant increase in the prevalence of mental health conditions among adolescents following lockdowns, jumping from 10.8% to 16.0%. This was coupled with reports of reduced sleep quality, loneliness, and fear. These studies are highly informative and suggest a strong shift toward negative mental health outcomes as a result of the pandemic. However, such self-report measures do not directly address the biological impact of stress on an individual. That is, perceived changes in psychological distress do not necessarily imply specific physiological mechanisms underlying changes in mood and behavior (Karlén et al., 2011). Researchers have formally called for longitudinally-measured physiological data to better understand the long-term impacts of the pandemic on mental health (Dantzer et al., 2020; Wade et al., 2020).

One potential target for assessing changes in physiological stress during the pandemic is measuring cortisol, a neuroendocrinological marker of stress (Burnard et al., 2017; Gibb et al., 2020). Research has frequently linked variability in cortisol to aspects of mood, behavior, and even brain morphology in developing youth (Joos et al., 2019; Miller et al., 2007; Russell et al., 2012). Generally, cortisol concentrations tend to increase in response to acute stressors, and basal levels of cortisol can become dysregulated in cases of chronic stress (Miller et al., 2007; Russell et al., 2012; Stalder et al., 2017). Greater increases in cortisol in response to a stressor (i.e., larger increases from basal levels following stress exposure) tend to be associated with greater perceived psychological distress, including increasing negative affect, depressive and anxiety symptoms, and post-traumatic stress (Phan et al., 2020; Stalder et al., 2017; Steudte-Schmiedgen et al., 2015).

Longitudinally measuring changes in circulating cortisol surrounding uncontrollable or unforeseen events was once nearly impossible because measurements are most commonly taken from saliva or blood (serum), both of which are highly time-sensitive and can rapidly change over the course of just a few minutes. Thus, to accurately measure, one would have to anticipate that the event was coming and take measurements immediately before and after. However, researchers have more recently begun to leverage the strength of measuring hair cortisol concentrations (HCC), as hair samples reliably encapsulate mean cortisol levels over time (Burnard et al., 2017; Lee et al., 2015). More specifically, hair grows at an average rate of 1 cm per month, thus each 1 cm segment of hair neatly catalogues the average concentration of circulating hormones over a one-month period (Short et al., 2016). These measurements are most reliable up to 5 cm from the scalp (Russell et al., 2012), thus researchers are able to retrospectively explore circulating hormones within an individual over an approximately 5 month period of time prior to biosample collection.

Critically, HCC has been repeatedly linked to mental health outcomes in adolescents and adults, though with some mixed findings. For example, a systematic review showed that major depressive disorder is associated with increased HCC, whereas anxiety disorders are generally characterized by decreased HCC, and post-traumatic stress disorder is denoted by an initial increase followed by a dramatic decrease in HCC to below baseline levels (Staufenbiel et al., 2013). Further, individual differences are known to impact the nature of HCC-psychopathology associations. Lehrer et al. (2020) demonstrated a robust link between greater perceived stress and elevated HCC, though this effect was modulated by resilience. Despite the heterogeneity in findings, there is a general consensus in the field that HCC is likely related to mental health outcomes and plays a critical role in the development of emotional neural circuitry, thereby setting the stage for risk and resilience to mental health outcomes across the lifespan (Kamin & Kertes, 2017).

Because HCC is able to historically catalogue physiological stress responses, it is possible to retrospectively and robustly assess changes in average cortisol concentrations surrounding natural, uncontrollable stressful events (Mewes et al., 2017; Steudte-Schmiedgen et al., 2015, 2016). For example, Gao et al. (2014) examined physiological stress following a major earthquake. Survivors exhibited significantly increased HCC shortly after the event, followed by a gradual decrease in HCC which finally normalized after approximately one year. In another study, Dajani and colleagues (2018) studied HCC among war-affected adolescents who were refugees displaced to another country. Youth presented with disparate patterns of cortisol dysregulation based on perceptions of mental health and personal safety. Specifically, HCC was elevated as a function of increasing perceived insecurity and exhibited significant dysregulation (i.e., unreliable change over time) among youth with greater post-traumatic stress disorder symptomatology. From these and other examples, it is clear that HCC is a promising approach for assessing physiological stress responses to uncontrollable events. Moreover, HCC can be linked to perceived changes in psychological well-being, providing scientists a powerful lens for probing the biological underpinnings of psychological distress following major stressors.

The present study focuses on physiological stress and perceived changes in mood among adolescents during the COVID-19 pandemic. We leveraged the historical sensitivity of HCC to assess changes in cortisol from pre-lockdown time periods to post-lockdown time periods. We expected that HCC would be significantly greater during post-lockdown periods relative to pre-lockdown periods. Additionally, we assessed retrospective perceptions of changes in mood, specifically positive and negative affect, prior to- versus since local lockdowns. Based on the extant literature, we hypothesized that youth would report increases in negative affect and decreases in positive affect during the pandemic. Finally, we assessed the extent to which perceived changes in affect and changes in HCC were related to one another. Because there is limited knowledge on the direction of effects, we examined three different associational models: 1) changes in affect and HCC are correlated, 2) changes in affect predict changes in HCC, and 3) changes in HCC predict changes in affect.

## Materials and Methods

### Participants

Children and adolescents who were enrolled in ongoing longitudinal studies of brain and cognitive development in youth (Stephen et al., 2021) were invited to participate remotely in the present sub-study following COVID-19 lockdowns. Only youths at the Omaha, NE site were contacted. In total, 63 youths aged 10- to 18-years-old (*M* = 15.02 years, *SD* = 2.02; 25 males) were enrolled in this sub-study probing the psychological repercussions of the COVID-19 pandemic. The study was completed between May 10, 2020 and June 20, 2020. Participants’ parents electronically signed informed consent forms for the sub-study, and youth participants electronically signed assent forms following a complete description of the protocol. All procedures were approved by the University of Nebraska Medical Center’s Institutional Review Board and met ethical standards in accordance with the Declaration of Helsinki.

### Questionnaires

Youth remotely completed a battery of questionnaires probing psychological repercussions of the COVID-19 pandemic (see Online Resources for details). Briefly, youth completed a portion of the “Emotional Experience” section of the COVID-19 Adolescent Symptom & Psychological Experience assessment (CASPE; Ladouceur, 2020), and a modified version of the Positive Affect Negative Affect Schedule – Child Form (PANAS-C; Hughes & Kendall, 2009; Laurent et al., 1999). These measures provided critical information on youths’ experiences of pandemic-specific stressors, and of their perceived positive and negative affect prior to- versus following local lockdowns.

### Hair Sample Collection

Parents were provided instructions for collecting hair samples and returning them by mail. All hair samples were taken between May 10, 2020 and June 20, 2020. Youth who declined to provide a hair sample (*n* = 9), and youth whose hair samples were less than 4 cm in length (*n* = 7) were excluded from analyses. For the purposes of this study, we focused on the most proximal (i.e., 0.0–1.0 cm from the scalp; “post-lockdown”) and the most distal (i.e., 3.0–4.0 cm from the scalp; “pre-lockdown”) hair segments to capture changes in cortisol concentrations prior to- versus post-lockdowns. Details on collection and assays are provided in Online Resources.

### Statistical Analysis

**Fig. 1:**
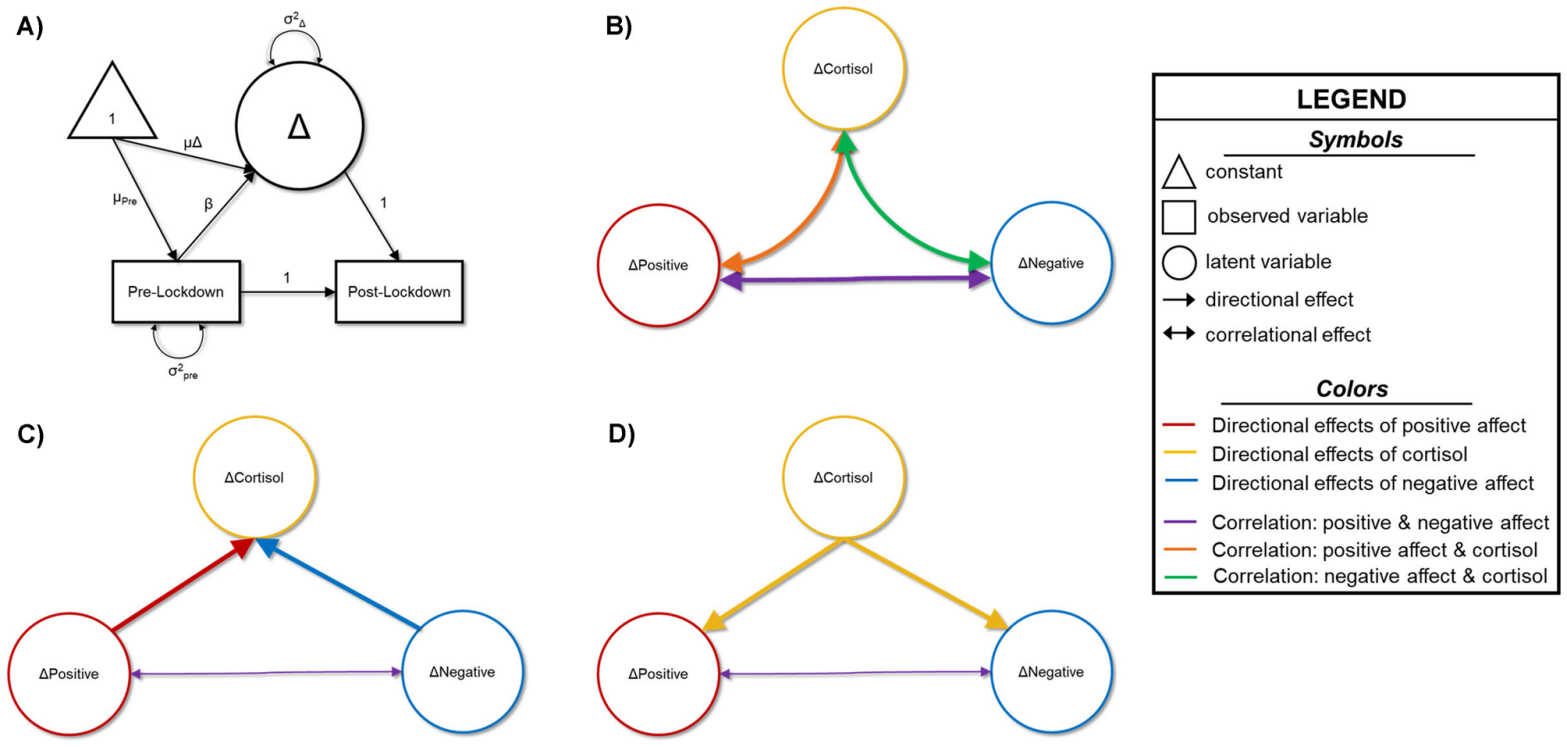
Conceptual Models. Conceptual figures depicting the basic models tested in the present study. For all models, lines in yellow indicate cortisol-specific associations/estimates in which cortisol is predicting another variable; lines in red are indicative of positive affect-specific associations/estimates in which positive affect is predicting another variable; lines in blue are indicative of negative affect-specific associations/estimates in which negative affect is predicting another variable; secondary colors (orange, purple and green) indicate associations that are non-directional (i.e., correlations); thicker lines highlight the main parameters involved in hypothesis testing. **A** The construction of the latent change score (e.g., ΔCortisol), defined by post-lockdown scores with the regression weight constrained to 1 (unstandardized). Pre-lockdown scores are predictors of the post-lockdown scores (constrained to 1), and of the latent change score (freely estimated; β). We freely estimate means (μ) and variances (σ^2^) of the pre-lockdown and latent change scores. A separate latent change score was defined for positive affect, negative affect, and hair cortisol concentrations. **B** Conceptual figure showing the correlational model of changes in positive affect, negative affect and cortisol. Double-headed arrows depict correlations. **C** Conceptual figure showing the model of changes in positive and negative affect predicting changes in cortisol. Double-headed arrows depict correlations, whereas single-headed arrows show directional predictive paths. **D** Conceptual figure showing the model of changes in cortisol predicting changes in positive and negative affect. Double-headed arrows depict correlations, and single-headed arrows show directional predictive paths

To assess the relationships between changes in HCC and changes in perceived negative and positive affect pre- to post-lockdown, we fit three variants of a latent change score model. Construction of the latent change variables was in accordance with accepted modeling parameters (details in Online Resources; see Fig. 1A; Kievit et al., 2018). We assessed model fit for each of the tested models (details in Online Resources). All models were computed in Mplus version 8.1.

#### Model 1: Correlational Model

We first examined a correlational model in which latent change variables of HCC, positive affect, and negative affect were allowed to freely correlate (Fig. 1B). In this variant of the model, we examined cross-domain coupling across all three measures. In other words, the latent change in cortisol, positive, and negative affect were all regressed on pre-lockdown cortisol, positive, and negative affect scores.

#### Model 2: Affect Predicts Cortisol

In the second variant of our analysis scheme, we modeled latent change in positive and negative affect as predictors of the latent change in cortisol (Fig. 1C). Latent change scores for positive and negative affect were allowed to correlate. With respect to cross-domain coupling, we removed the paths from pre-lockdown cortisol measures to latent changes in positive and negative affect given the directional predictions implied in the model.

#### Model 3: Cortisol Predicts Affect

In the final model, we examined the latent change in cortisol as a predictor of latent change in positive and negative affect (Fig. 1D). Cross-domain coupling paths linking pre-lockdown positive and negative affect to the latent change in cortisol were removed given the directional nature of the model. As before, latent changes in positive and negative affect were allowed to freely correlate.

## Results

### Final Sample for Analysis

Forty-seven youth had complete HCC and questionnaire data. Two youth had excessively high HCC measures, and one youth had an excessively high pre-lockdown negative affect score (values exceeding ± 2.5 standard deviations of the sample mean). Thus, the final sample was comprised of 44 youth age 10- to 18-years-old (*M* = 14.70, *SD* = 2.04; 13 male) who completed all measures between May 10, 2020 and June 18, 2020. Given the dates of hair sample collection, coupled with an average growth rate of 1 cm per month, the most distal segment of hair captured approximately mid-January to mid-February of 2020. This timeframe approximately spanned the one-to-two months prior to the onset of local lockdowns (see next section). Complete demographics are detailed in Online Resources Table S1.

### Contextual Information and Timeline of Local Events

The study took place in the greater Omaha, Nebraska area. The medical center downtown and its biocontainment unit began monitoring the novel coronavirus in mid-January, shortly before the first confirmed cases of COVID-19 in the U.S. (January 21). By mid-February, the U.S. government had declared a public health emergency, and Omaha became a host site for treating American evacuees from abroad who were infected with the disease. Nebraska saw its first positive COVID-19 case outside of the biocontainment center on March 6 amid reports of panic shopping and empty store shelves. Over the following week, the World Health Organization declared COVID-19 a pandemic (March 11), the U.S. declared COVID-19 a national emergency (March 13), and public schools in the Omaha area closed their doors (March 13–19). Local stores, restaurants, childcare facilities, and recreational areas were mandated to close shortly thereafter. Unemployment claims skyrocketed almost overnight. Tensions in the local community rose over the next month (April) as governing officials sent mixed messages and began lifting restrictions. Several nearby cities became hotspots for COVID-19 due to working conditions (e.g., meat packing plants), but employees were forced to continue working or risk termination. The Omaha city mayor mandated that city parks be closed only two weeks before the state governor announced plans to walk back restrictions on stores and restaurants. In early May, Omaha businesses began reopening at limited capacity with masking and social distancing guidelines. Over the next few weeks, restrictions continued to ease and Omaha hospitals reported all-time high numbers of COVID-19 patients. Nebraska saw its first cases of multisystem inflammatory syndrome in children, a condition impacting youths who were previously infected with COVID-19, in early June. For more, see Ryan (2021).

### Characterization of Pandemic- and Lockdown-Related Stressors

Overall, 97.73% of youth in the present study indicated that at least one pandemic- or lockdown-specific stressor was concerning them in the past seven-days (*M* = 7.73 stressors, *SD* = 3.70). Over one-third of the sample (36.36%) rated at least one stressor as concerning them “a lot” or “a great deal” (i.e., a score of 4 or 5). Descriptive statistics for each item are reported in Table 1. We also examined participants’ open-ended responses in which they could write down any additional concerns over the past seven days that were not covered in the questionnaire. Nearly two-thirds of participants (63.64%) reported additional concerns. We qualitatively coded major themes among the open-ended responses, and the most common threads were related to concerns over one’s personal future (e.g., returning to school/difficulties getting in to college, concerns with with returning to work; 32.14% of responses), chronicity of the pandemic (28.57% of responses), and concerns about maintaining friendships (25.00% of responses). Examples of free-form responses follow:

*“I'm worried that I could cause someone to get sick, and have to live with the guilt. Worry about friendships disintegrating. Worry that life will NEVER return to normal, worry that I'll never get a hug again.” – age 14*

*“that this will continue for a lot longer than we expected and it will change the course of my life” – age 15*

*“I missed graduation, I haven't seen my friends or talked to them at all and babysitting my brothers is terrible.” – age 14*

*“If it's ever gonna end, what it's gonna be like when and if it ends.” – age 12*

**Table 1:** Descriptive statistics for level of concern over the past seven days for each of 16 unique pandemic- and lockdown-related stressors

	**Concern**		**Endorsement**	
**Stressor**	***M***	***SD***	***N***	**%**
Not seeing friends in person	2.93	1.40	34	77.27%
Family might get sick	2.68	1.31	34	77.27%
People might die if they get sick	2.59	1.28	34	77.27%
Friends might get sick	2.43	1.26	31	70.45%
Miss events that were important to me (e.g., graduation)	2.36	1.42	27	61.36%
I might get sick	1.95	1.14	24	54.55%
Having to stay home	2.07	1.21	23	52.27%
Sibling conflicts	1.84	1.03	21	47.73%
Not having enough money	1.84	1.18	19	43.18%
Parents will lose their jobs	1.82	1.11	19	43.18%
Having to spend more time with family	1.66	1.01	18	40.91%
Conflict with parents	1.57	1.02	14	31.82%
Falling behind with schoolwork	1.61	1.20	12	27.27%
Not getting into college	1.48	1.07	11	25.00%
Conflict between parents	1.41	0.84	10	22.73%
Having enough to eat	1.36	0.78	9	20.45%

	***M***	***SD***	***Range***	
Number of stressors endorsed	7.73	3.70	0–16	
Number of events rated ≥ 4	0.98	1.69	0–6	

*N* and % indicate the number and percentage of the overall sample of youth who endorsed that specific stressor (score > 1). Ratings of concern over the past seven days were completed using a 5-point Likert scale where: 1 = very little/not at all; 2 = a little; 3 = some; 4 = a lot; 5 = a great deal

### Descriptive Statistics for HCC and Affect

Characteristics of included participants’ hair types and hair treatments are reported in Online Resources (Table S2). Descriptive statistics for the measures of interest are shown in Online Resources Fig. S1 and Table S3. In general, HCC (*t*(46) = -2.85, *p* = 0.007) and negative affect (*t*(46) = -2.15, *p* = 0.04) increased between pre- and post-lockdown periods, whereas positive affect did not significantly change (*t*(46) = 0.66, *p* = 0.51). We report additional exploratory follow-up analyses probing changes in affect related to pandemic-specific stressors in Online Resources. Correlations between all variables of interest are listed in Online Resource Table S4.

### Model 1: Correlational Model

**Fig. 2:**
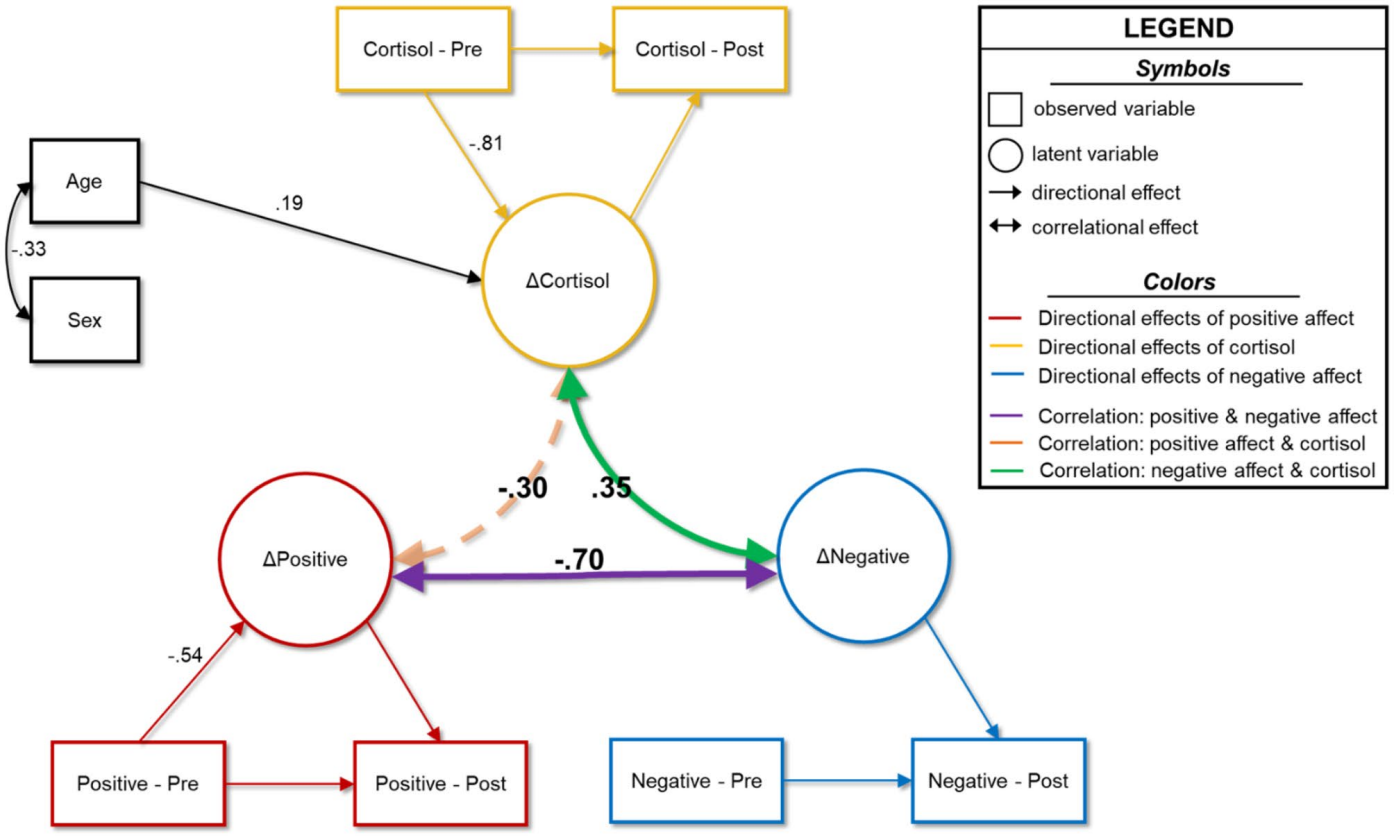
Correlational model of HCC and affect. Results of the correlational model examining relationships between latent changes in hair cortisol concentrations, positive affect, and negative affect. Age and sex serve as control variables. Double-headed arrows show correlations, and single-headed arrows show directional predictive relationships. Dashed lines indicate non-statistically significant relationships, whereas solid lines show significant associations at the *p* < 0.05 level. Only statistically significant parameters and parameters central to testing our hypotheses are shown in for simplicity. All reported coefficients are standardized. Complete model results are shown in Online Resources Fig. S2

The correlational model results are shown in Fig. 2 and Online Resource Fig. S2. Model fit indices are detailed in Online Resource Table S5. Turning first to cortisol measures the latent change in cortisol was negatively associated with pre-lockdown measures (β = -0.81, b = -0.78, 95% CI[-0.94, -0.63], *p* < 0.001), suggesting that those who had high cortisol to begin with saw less robust increases (or possibly saw decreases) following lockdowns. Additionally, older youth tended to have greater increases in cortisol from pre- to post-lockdown periods (β = 0.19, b = 0.37, 95%CI[0.05, 0.68], *p* = 0.021). There was no significant association with sex.

Looking at perceived affect assessed by the PANAS-C, the latent change in positive affect was negatively associated with pre-lockdown scores, such that youth who retrospectively reported higher positive affect prior to lockdowns tended to have greater decreases in positive affect following lockdowns (β = -0.54, b = -0.69, 95%CI[-1.04, -0.34], *p* < 0.001). Interestingly, there was no significant association between retrospectively-reported pre-lockdown negative affect and the degree of change in negative affect (β = 0.28, b = 0.23, 95%CI[-0.007, 0.47], *p* = 0.12). Neither age nor sex was significantly associated with the latent change in affect scores. There were no significant cross-domain predictions in the model.

Finally, correlations among the latent change scores showed that changes in HCC were significantly associated with changes in negative affect (ρ = 0.35, b = 4.04, 95%CI[0.44, 7.65], *p* = 0.028). The association between changes in cortisol and changes in positive affect was trending toward significance (ρ = -0.30, b = -4.60, 95%CI[-9.40, 0.19], *p* = 0.06). These data suggest that youth with greater increases in HCC tended to report greater increases in negative affect from pre- to post-lockdown and trended toward reporting greater decreases in positive affect from pre- to post-lockdown. Finally, the latent change in negative affect was also associated with the change in positive affect (ρ = -0.70, b = -31.67, 95%CI[-47.93, -15.41], *p* < 0.001). Thus, youth who tended to have greater increases in negative affect also tended to have greater decreases in positive affect.

### Model 2: Affect Predicts Cortisol

**Fig. 3:**
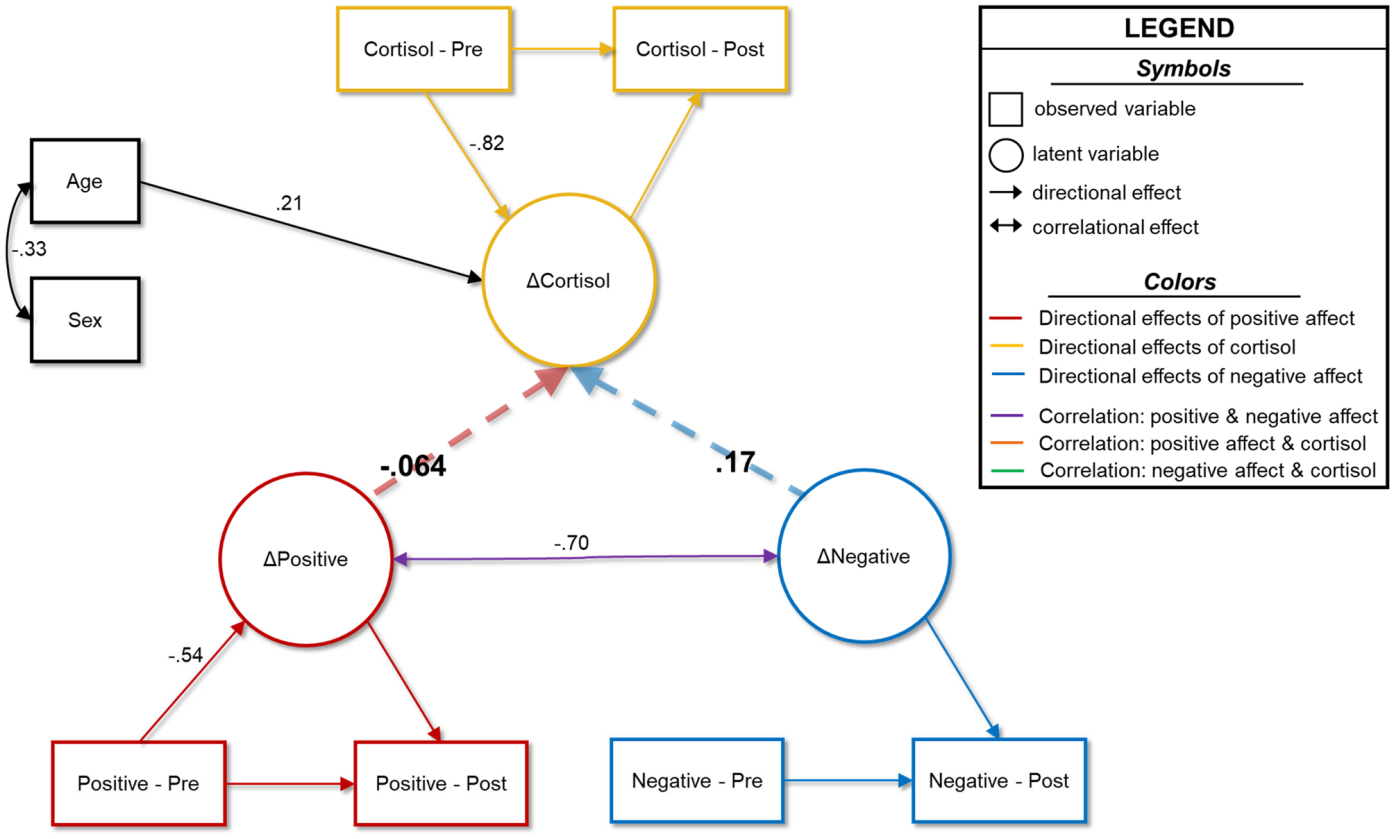
Predictive model wherein affect predicts cortisol. Results of the model in which changes in positive and negative affect are predictors of changes in hair cortisol concentrations. Age and sex serve as control variables. Double-headed arrows show correlations, and single-headed arrows show directional predictive relationships. Dashed lines indicate non-statistically significant relationships, whereas solid lines show significant associations at the *p* < 0.05 level. Only statistically significant parameters and parameters central to testing our hypotheses are shown in for simplicity. All reported coefficients are standardized. Complete model results are shown in Online Resources Fig. S3

The model in which latent changes in affect scores predicted the latent change in HCC had excellent fit, though the change in AIC and BIC relative to the correlational model was minimal (Table S5). The conclusions regarding latent change score construction, cross-domain coupling, and relationships to age and sex remained the same (Fig. 3; Online Resources Fig. S3). Likewise, the latent change scores of positive and negative affect were still strongly correlated (ρ = -0.70, b = -31.92, 95%CI[-48.30, -15.54], *p* < 0.001), suggesting that youth with greater increases in negative affect tended to see greater decreases in positive affect. Interestingly, neither of the predictions from latent change in affect scores to latent change in cortisol was significant (*p*’s = 0.15 and 0.63).

### Model 3: Cortisol Predicts Affect

**Fig. 4:**
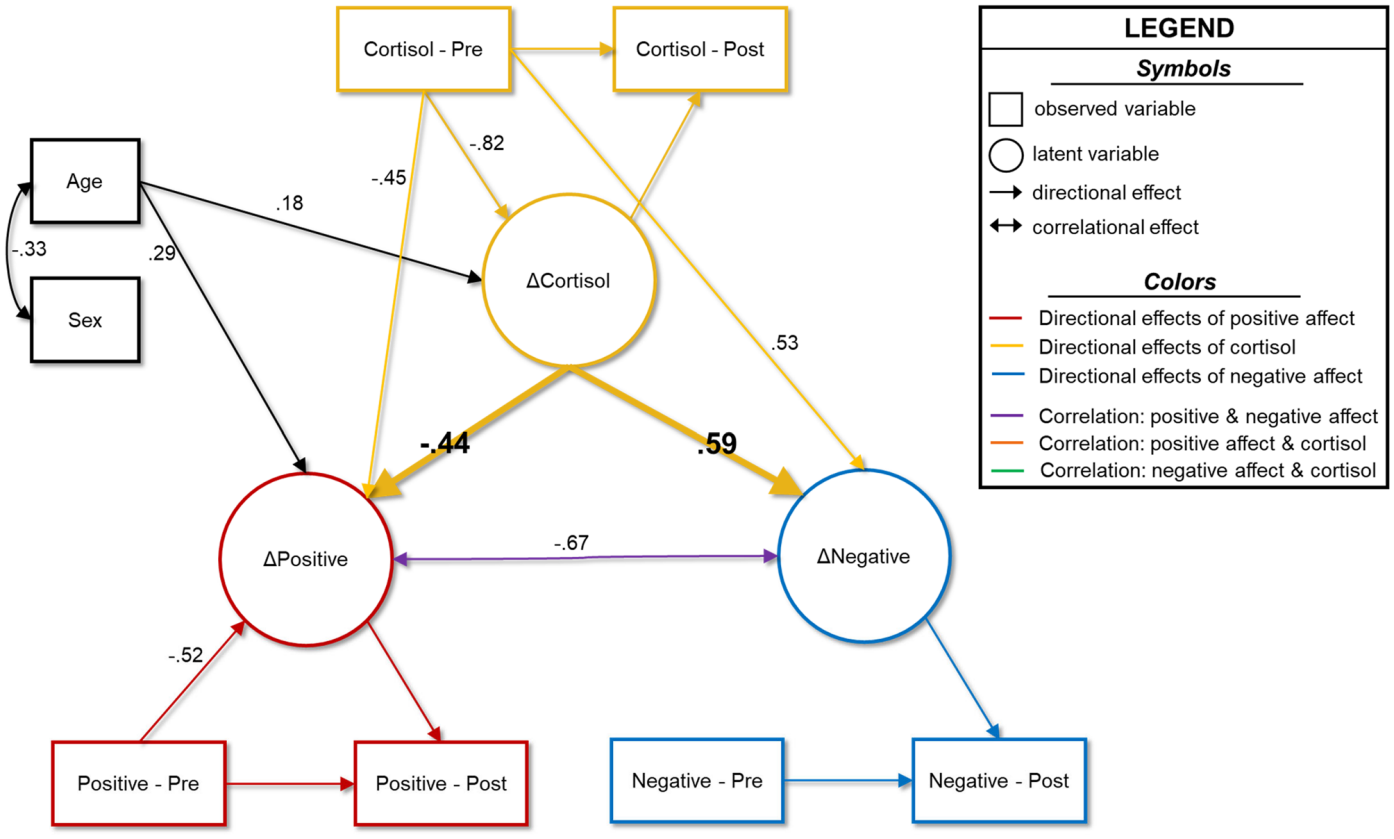
Predictive model wherein cortisol predicts affect. Results of the model in which changes in hair cortisol concentrations predict changes in positive and negative affect. Age and sex serve as control variables. Double-headed arrows show correlations, and single-headed arrows show directional predictive relationships. Dashed lines indicate non-statistically significant relationships, whereas solid lines show significant associations at the *p* < 0.05 level. Only statistically significant parameters and parameters central to testing our hypotheses are shown in for simplicity. All reported coefficients are standardized. Complete model results are shown in Online Resources Fig. S4

Our final model examined whether latent change in cortisol could predict latent changes in positive and negative affect. Again, the model had excellent fit, and showed minimal change in fit compared to the correlational model (Table S5). Conclusions regarding latent change score construction, cross-domain coupling, and relationships to age and sex remained largely the same, with a few key exceptions (Fig. 4; Online Resources Fig. S4). There was evidence of significant cross-domain coupling such that pre-lockdown HCC was significantly associated with latent changes in both positive (γ = -0.45, b = -1.13, 95%CI[-2.17, -0.09], *p* = 0.034) and negative affect (γ = 0.59, b = 0.87, 95%CI[0.12, 1.62], *p* = 0.024). Thus, in this model, the data suggested that youth who had higher HCC pre-lockdown tended to have greater decreases in positive affect, and greater increases in negative affect from pre- to post-lockdown. Additionally, we detected a significant association between age and the latent change in positive affect (β = 0.29, b = 1.44, 95%CI[0.18, 2.69], *p* = 0.025), with older youth tending to report less of a decrease in positive affect over time relative to younger participants. Importantly, changes in HCC were robustly predictive of both changes in positive affect (α = -0.44, b = -1.16, 95%CI[-2.26, -0.05], *p* = 0.040) and changes in negative affect (α = 0.59, b = 1.02, 95%CI[0.22, 1.82], *p* = 0.013). The data suggest that youths who showed greater increases in cortisol over time tended to report greater increases in negative affect, and greater decreases in positive affect.

## Discussion

The present study examined the extent to which physiological stress responses and mood changed during the COVID-19 pandemic in typically-developing children and adolescents. As expected, youth reported significant increases in negative affect after lockdowns began. Contrary to our hypothesis, there was no significant shift in positive affect overall, though we did see an effect in youth who reported greater levels of recent concern over pandemic-related stressors. Additionally, we saw significant increases in mean HCC following local lockdowns, suggesting robust physiological stress responses surrounding the pandemic. Our key finding was that changes in HCC robustly predicted perceived changes in mood. Notably, changes in mood did *not* predict changes in HCC. We further discuss these findings and their implications below.

The greatest strength of this study was the measurement of HCC prior to and after local lockdowns began. By using HCC, we were able to reliably index shifts in circulating cortisol levels among youth surrounding a pivotal, high-stress period of the COVID-19 pandemic. As expected, we saw robust increases in HCC following local pandemic-related lockdowns. Moreover, the degree of change in circulating cortisol was dependent on baseline levels, such that youth who had higher HCC prior to lockdowns tended to show smaller increases in circulating cortisol than youth with lower baseline HCC. Our findings were well-aligned with the extant literature showing increases in cortisol following a major stressful event (e.g., Dajani et al., 2018; Gao et al., 2014). Additionally, our data corroborate previous findings suggesting that individuals with lower relative baseline cortisol levels tend to show greater increases following a stressor (Joos et al., 2019; Steudte-Schmiedgen et al., 2015).

These data uniquely contribute to our knowledge of pandemic-related stress from a neuroendocrinological approach. Notably, physiological stress responses are markedly different depending on the nature of the stressor, and researchers have yet to characterize shifts in circulating cortisol levels surrounding a global pandemic. Meta-analyses and large-scale reviews of physiological stress responses have shown that stressful events which threaten the physical self (e.g., bodily harm/war) versus the social self (e.g., divorce) have very different presentations (Miller et al., 2007; Russell et al., 2012; Stalder et al., 2017). Additionally, uncontrollability, unpredictability, chronicity, and perceived trauma and psychological distress each contribute to physiological responses. The COVID-19 pandemic is unique relative to many other studied catastrophic events given the multiplicative types of stressors it presents. For instance, there are threats to the physical self (e.g., falling ill) *and* to the social self (e.g., loss of friendships/social supports), unpredictability and uncertainty surrounding the progression and duration of the global spread (e.g., poor government organization and communication), and psychological distress and disruptions to daily life due to lockdowns (Courtney et al., 2020). This is compounded by the potential for other forms of harm, such as reduced access to healthcare and social services, and increased risk of child abuse in the home going unnoticed (Fisher et al., 2020; Park et al., 2020; Patrick et al., 2020).

In the current study, we also explored the extent to which shifts in HCC and affect were related to one another. Changes in cortisol and affect were all well correlated, which was in accordance with prior literature (e.g., (Peeters et al., 2003). We found that changes in HCC robustly predicted increases in negative affect and decreases in positive affect, though affect did not significantly predict physiological stress responses. These data suggest that physiological stress responses may, at least in part, drive changes in affect, though this requires further investigation. If these findings are replicable in larger, more sociodemographically diverse samples, they could allude to potential avenues for intervention or prevention of negative mental health outcomes. Namely, pharmacologic regimens that help regulate or balance hormone levels may tamp subsequent maladaptive psychological impacts following major stressful events. Others have similarly suggested that dysregulated levels of circulating cortisol over prolonged periods may cause new or worsening psychological symptoms, even in otherwise “healthy” individuals (Staufenbiel et al., 2013).

One potential avenue by which elevated cortisol may contribute to increasing psychological symptoms is through its impacts on brain structure and function. Ulrich-Lai and Herman (2009) detailed the manner in which limbic regions of the brain, which span cortical and subcortical areas, contribute to the homeostatic balance in neuroendocrine and autonomic functioning in day-to-day life. However, under times of extreme or prolonged stress, cortisol secretions become dysregulated and actually cause damage to these neural structures via dendritic modifications and atypical expression of neurotransmitter receptors. These fundamental shifts in physiology affect subsequent neural functioning and in turn, psychological well-being, including anxious and depressive symptomatology (Gillespie & Nemeroff, 2005; Ulrich-Lai & Herman, 2009). It is possible that, in the case of the COVID-19 pandemic, the prolonged stress of local lockdowns and fear for the health of oneself and loved ones may be gradually affecting neural structure and function in these youth, thereby leading to poorer psychological wellbeing. However, further work is needed to determine the extent to which brain structure and function may have been altered during the current pandemic. This would be an excellent area of research for those with access to large longitudinal datasets with pre-pandemic baseline measures available.

Without a doubt, the COVID-19 pandemic is a unique phenomenon that warrants continued investigation of prolonged stress responses and changes over time. Presently, these data provide a window into the underlying physiological stress responses surrounding adolescents’ experiences early in the pandemic. Other recently published works have detailed prolonged self-reported psychological outcomes among youths as the pandemic has continued through additional waves. For instance, Ravens-Sieberer et al. (2021) assessed mental health among children and adolescents in Germany during the first wave of the pandemic, and again almost one year into the pandemic. The study found generally increased reports of depressive and psychosomatic symptoms at the second measurement point, though risk (e.g., social disadvantage) and resilience factors (e.g., positive family environment) certainly moderated these outcomes. Further investigation should follow up with youths and assess the degree to which prolonged neuroendocrinological dysregulation is associated with these mental health outcomes.

Our data are consistent with a growing body of literature reporting robust increases in psychological distress and negative mood during the pandemic in a multitude of populations (Duan et al., 2020; Hyland et al., 2020; Marques et al., 2020; Rogers et al., 2021). It is rather intuitive that negative affect would have significantly increased given the circumstances of the lockdowns. The pandemic forced municipalities across the country to close schools and businesses, restrict access to public areas like parks, and recommend that individuals confine interpersonal interactions to members of their own households (Coyne et al., 2020; Fisher et al., 2020). These burdens pervasively impacted youth in the present study as evidenced by reported levels of recent concern. An overwhelming majority of youth indicated that at least one pandemic-related stressor had concerned them in the past week, with an average of roughly seven-to-eight endorsed stressors per participant. Stress burden is known to concomitantly increase with psychological distress and negative mood (Mohler-Kuo et al., 2021; Park et al., 2020; Rajkumar, 2020; Taylor, 2019).

Contrary to our hypothesis, we did not see changes in positive affect in the sample overall. Rather, we found that significant decreases in positive affect were specific to youth who reported a greater degree of recent concern over pandemic-related stressors. This finding critically identifies youth who may be more at risk of suffering negative mental health outcomes due to the pandemic. Positive affect has long been associated with resilience in the face of adversity; those who report greater positive affect tend to have better mental and physical health outcomes, even in light of major stressors like life-changing medical diagnoses (Lord et al., 2015; Murphy et al., 2017). Thus, it is possible that youth in the current study who experienced greater concern and greater decreases in positive affect may be less resilient to, and more affected by, the stress induced by the COVID-19 pandemic. These youth may be prone to anxious or depressive moods, and thus may benefit from targeted services that provide more structured support to reduce concerns. For example, safe access to social support networks and improved approaches to online or hybrid learning could help alleviate concerns with maintaining friendships and returning to school (e.g., Magson et al., 2021).

The present study is not without limitations. First, affect was measured retrospectively, whereas HCC was assessed longitudinally making it difficult to equivalently compare temporal relationships. Ideally, researchers would have baseline measures of typical affect prior to the onset of a stressful event; however, this is a common shortcoming of research focused on the impacts of unpredictable catastrophic events. Large-scale studies like the Adolescent Brain Cognitive Development (ABCD) study (Casey et al., 2018), which collect longitudinal measurements of emotional, mental, and physical health, could be critical in affording the field solid baseline measurements of individuals in the event of unpredictable major stressors. Second, the sample in the current study was relatively small and sociodemographically homogenous, and may not be representative of the greater diversity of youth across the country. Further efforts should be taken to study socioeconomically and demographically diverse individuals across multiple geographic regions to better characterize the effects of the pandemic. In particular, capturing variability in HCC among males, for whom our sample was very limited due to the long hair length requirements, would be beneficial. In addition, testing our proposed latent change score models in larger samples would be ideal. It is a common misconception that such models require large (*N* > 200) samples to estimate properly (Iacobucci, 2010; Kim, 2005; Marsh et al., 2004; Wolf et al., 2013). That said, testing the models in larger and more diverse samples would address the generalizability of our findings to the broader public. Finally, for our hair samples, we relied on the notion that hair grows an average of 1 cm per month. Hair growth rates may vary from person-to-person (Burnard et al., 2017), and our presumed timeframes captured by the different hair segments may vary slightly between individuals.

### Conclusions

The COVID-19 pandemic is an unprecedented global stressor that has had far-reaching impacts on physiological and mental health. Our study of adolescents during the first wave of the pandemic revealed significant increases in physiological stress surrounding local lockdowns beginning in March of 2020. Specifically, we found significant increases in HCC from pre- to post-lockdown time periods. Importantly, those increases in cortisol were robustly predictive of changes in perceived positive and negative affect surrounding lockdowns. The present study highlights the severity and impact of the COVID-19 pandemic on youth and supports the need for resources for youth coping with the vast fallout of this globally catastrophic event.

## Supplementary Information

Below is the link to the electronic supplementary material.

Supplementary file1 (DOCX 907 KB)
