# Supplementary material for: Increases in Circulating Cortisol during the COVID-19 Pandemic are Associated with Changes in Perceived Positive and Negative Affect among Adolescents
Source: Res Child Adolesc Psychopathol. 2022 Sep 1;50(12):1543–55. doi: 10.1007/s10802-022-00967-5 (PMC9435427; doi:10.1007/s10802-022-00967-5)
Supplement: Supplementary file 1 — Supplementary file1 (DOCX 907 KB) [file 10802_2022_967_MOESM1_ESM.docx]

**Supplemental Methods**

**Questionnaires**

All questionnaires were delivered electronically via the Collaborative Informatics and Neuroimaging Suite (COINS; Landis et al., 2016; Scott et al., 2011), and participants could complete the surveys at their leisure within a one-week span. Youth completed a portion of the “Emotional Experience” section of the COVID-19 Adolescent Symptom & Psychological Experience assessment (CASPE; (Ladouceur, 2020)). In this section, youth were asked to rate the level of concern they felt about 16 pandemic- and lockdown-relevant stressors in the past seven days (e.g., not seeing friends in person, family might get sick). Ratings were completed on a 5-point Likert scale (1 = *very little or not at all*, 5 = *a great deal*). Youth were also given an open-ended response box to record any stressors that had been affecting them in the past seven days that were not already included in the list.

Youth also completed an adapted version of the Positive Affect Negative Affect Schedule – Child Form (PANAS-C; Hughes & Kendall, 2009; Laurent et al., 1999). The traditional PANAS-C is comprised of 30 items related to positive (e.g., proud, calm) and negative affect (e.g., nervous, ashamed). Youth were instructed to indicate to what degree each affective descriptor captured their feelings in the past few weeks on a 5-point Likert scale (1 = *very slightly or not at all*, 5 = *extremely*). For the present study, we adapted the questionnaire to probe perceptions of affect prior to- versus since local lockdowns. The instructions for the survey read as follows:

*“This scale consists of a number of words and phrases that describe different feelings and emotions. Read each item and select the appropriate answer for that word. Indicate how much you felt that way 1) in the 6 months prior to when people started staying home (about March 15, 2020), and 2) since people started staying home (since about March 15, 2020).”*

Thus, the modified measure allowed us to assess participants’ retrospective perceptions of their affect pre- and post-lockdowns. We computed sum scores for positive and negative affect for each timeframe to assess potential changes in affective experiences surrounding the pandemic. Five youth did not complete the PANAS-C and were excluded from further analyses. Of note, the PANAS has excellent validity and has been linked to post-traumatic stress and trauma in the literature. The negative affect scale is commonly and robustly associated with symptoms such as externalizing behaviors, avoidance, emotional numbing (Monson et al., 2004; Raudales et al., 2019; Seligowski & Orcutt, 2016). Conversely, the positive affect scale is frequently inversely associated with aspects of negative mood like anhedonia (Seligowski & Orcutt, 2016), and positively associated with resilience factors like positive emotionality (Contractor et al., 2019; Frewen et al., 2012).

Finally, parents completed the Barratt Simplified Measure of Social Status (BMSS; (Barratt, 2006) in order to approximate household socioeconomic status. Total scores can range from 17 to 66, with higher scores indicated higher socioeconomic status.

**Hair Sample Collection and Assay**

Parents were provided with video instructions via a web-based platform and paper copies of instructions with pictures, collection materials (e.g., clips, scissors, foil, ID labels, padded envelope), and a brief questionnaire probing hair type and hair treatments (Short et al., 2016). Parents were instructed to comb, tie, and cut a small section of hair totaling approximately 100 strands as close as possible to the scalp from the posterior vertex (i.e., back of the head). Samples were placed unbent into an aluminum pouch, sealed with an ID label, placed into a padded envelope, and shipped to the Iowa State University Stress Physiology Investigative Team Laboratory where they were stored in the dark at room temperature. Home-collection has been validated for hair samples in adults and adolescents (Ouellet-Morin et al., 2016; Ramirez et al., 2017).

Hair was segmented into four sequential 1cm sections. Given that the average growth rate for hair is 1 cm per month (Wennig, 2000), coupled with the timing of hair sample collections for the present study relative to pandemic-related lockdowns, we analyzed four total 1 cm segments to capture hormone concentrations among youth over the past four months straddling the start of local lockdowns. For the purposes of this study, we focused on the most proximal (i.e., 0.0-1.0 cm from the scalp; “post-lockdown”) and the most distal (i.e., 3.0-4.0 cm from the scalp; “pre-lockdown”) hair segments to capture changes in cortisol concentrations prior to- versus post-lockdowns. Following standard protocols (Short et al., 2016; Wang et al., 2016, 2019), each segment was washed twice in 2.5 ml of isopropanol for 3 minutes to eliminate impurities using a rotator. Samples were dried for 2 days under a fume hood and then ground to a fine powder in a ball mill for 6 minutes at a speed of 30 Hz. To reduce sample loss, each sample was ground within the same cryovial as used during extraction. Samples of powdered hair were weighed on an analytic balance and then extracted with 1.5ml methanol. Samples were incubated for 18-24 hours at room temperature with constant inversion using a rotator. Next, the samples were centrifuged at 5000 rpm for 5min to pellet the powdered hair. The supernatant was transferred to a new cryovial and, if necessary, aliquoted for multiple assays. Samples were dried down using a nitrogen evaporator at 50°C for 20 minutes. The steroid extract was then reconstituted using assay diluent. This reconstituted sample was assayed using commercially available enzyme-immunoassays (www.salimetrics.com). Inter-assay variability was 3.73% (control highs = 3.09%, control lows = 4.36%), and the overall intra-assay variability was 9.50% (most proximal segment = 7.35%, most distal segment = 11.65%), which was within the acceptable range of error (Tworoger & Hankinson, 2006).

**Statistical Analysis: Latent Change Score Models**

The current study employed latent change score models to address our specific research questions: 1) to what degree did mental wellness and circulating cortisol change from a pre-lockdown time to post-lockdowns, and 2) how are these changes (specifically magnitude of change) related to one another? Using the latent change score approach, we leveraged difference scores per domain (i.e., cortisol, positive affect, and negative affect) to understand the degree to which the amount of change over time in a specific domain was dependent on pre-lockdown scores in all domains (i.e., cross-domain coupling), which is far more representative of the known nature of endocrine-mental health associations than more basic statistical approaches afford. Further, we were able to explore all of this while probing our primary research question regarding directionality of effects (i.e., do changes in cortisol predict changes in affect or vise versa), while concurrently controlling for demographic characteristics known to influence both mental health and endocrinology during adolescence. Given the desire to model changes over time, and to enter those changes over time into a more complex model, the latent change score technique was favorable over other similar approaches like linear mixed effects modeling (Kievit et al., 2018; McNeish & Matta, 2018).

For each of the three measures of interest, the post-lockdown measure was regressed on the pre-lockdown measure with the regression weight constrained to 1. We then defined a latent change variable using the post-lockdown measure with the regression weight constrained to 1. This effectively forced all of the variance from the observed variable, after accounting for the pre-lockdown measure association, into the latent change score. The remaining means and variances for the observed post-lockdown measure were constrained at 0 because there should be no remaining variance in the observed variable after constructing the latent change score. The latent change variable was regressed on the pre-lockdown measure. All other parameters were freely estimated. Pre-lockdown measures were all allowed to freely correlate. As appropriate, we examined cross-domain coupling. In each model, all three latent change variables were regressed on age and sex to account for potential developmental effects. Relationships among latent change variables are described in the main text and varied across models. Results reported include standardized regression coefficients, which were computed in Mplus using the “STDYX” option in the “OUTPUT” command (b_StdYX_ = b*SD(x)/SD(y); Muthen & Muthén, 2012).

**Model Fit**

We assessed model fit for each of the three models of interest based on model fit criteria outlined by Hu and Bentler (1999). Good model fit was determined by a root mean squared error of approximation (RMSEA) < .06, a comparative fit index (CFI) > .95, and a standardized root mean square (SRMR) < .08. We also assessed the chi square statistic, wherein a non-significant statistic indicates good fit of the model to the data. Finally, we report both Akaike and Bayesian Information Criteria (AIC and BIC, respectively) to compare fit across models. Shifts in AIC and BIC > 10 are considered robust shifts in model fit, with lower values suggesting better fit.

**Supplemental Results**

**Exploratory Follow-Up Analysis**

As stated in the main text, we did not detect any significant differences in perceived positive affect before versus after local lockdowns began, though there appeared to be significant

variability in the sample. This variability may have been the result of differences in perceived stress or level of concern about the pandemic in general. In other words, youth who were less

concerned about the pandemic may have been less likely to note significant changes in affect during the pandemic. Thus, we created a dichotomous grouping of “high concern” versus “low concern” based on responses to the CASPE questionnaire. Youth who responded to one or more items on the CASPE with a score of at least 4 (4 = *a lot*; 5 = *a great deal*) comprised the “high concern” group (*n* = 16), and the rest of youth were in the “low concern” group (*n* = 28). Using extracted latent change scores for positive and negative affect and for HCC, we ran three separate hierarchical regressions to determine whether those changes were related to level of concern about pandemic-related stressors. The first level of the model included control variables of age and sex. The second level of the regression included our newly-created dichotomous grouping variable of low- versus high-concern (coded 0 = *low*, 1 = *high*). For completeness, we included a third level with the total number of stressors endorsed in the CASPE as a predictor variable. This allowed us to determine whether the *severity* or the *quantity* of stressors may play a more significant role in changes in HCC and affect. We conservatively controlled for type I error inflation using a Bonferroni correction for the three regressions (α = .017).

For the model of changes in positive affect, adding the grouping variable of low- versus high-concern significantly improved model fit (Δ*R*^2^ = .14, *p* = .006), but adding in the total number of stressors endorsed did not (Δ*R*^2^ = .00, *p* = .98). Thus, the best fitting model included age, sex, and low- versus high-concern group (*R*^2^ = .29, *F*(3, 40) = 6.85, *p* = .001). Only the concern group variable significantly predicted changes in positive affect in the present model given our conservatively corrected α-level. Youth who were grouped as high-concern tended to report significant decreases in positive affect (β = -.39, *p* = .006). Similarly, the model of changes in negative affect was significantly improved by adding in the concern grouping variable (Δ*R*^2^ = .14, *p* = .006), but not the total number of stressors (Δ*R*^2^ = .00, *p* = .94). The best fitting model included age, sex, and concern group (*R*^2^ = .32 *F*(3, 40) = 6.13, *p* = .002). Again, only concern group was a significant predictor of changes in affect given our conservative threshold, with those labeled as high concern showing significant increases in negative affect from pre- to post-lockdown periods (β = .39, *p* = .006). Finally, the model of changes in HCC showed no significant effects, and none of the models were significant (overall model *p*’s = .16 to .28).


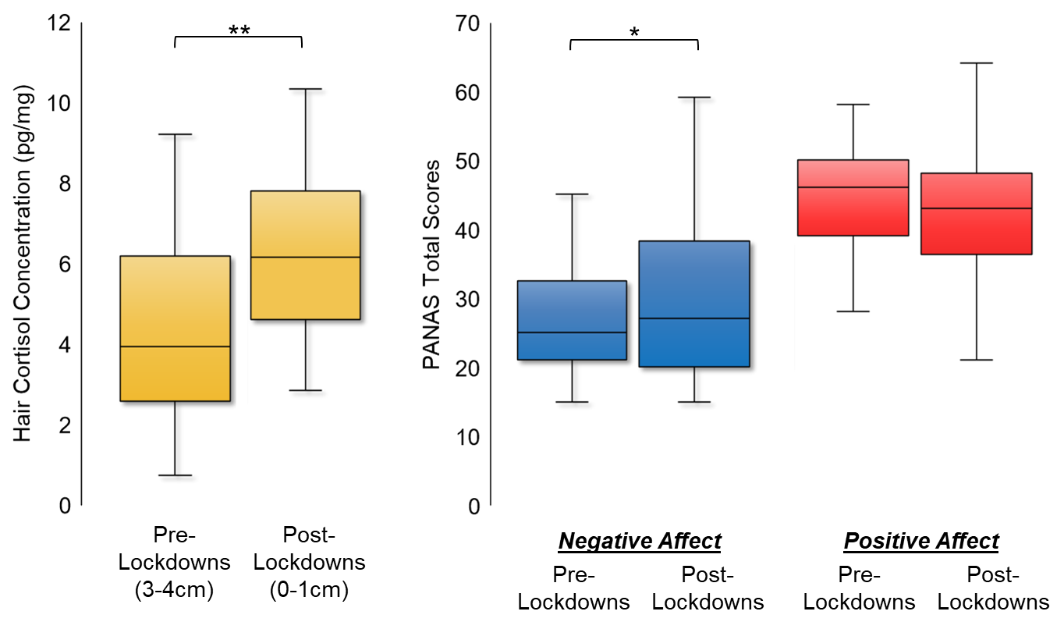


***Figure S1.*** **Box plots of pre- and post-lockdown measures**

Box plots showing the distribution of pre- and post-lockdown measures of hair cortisol concentrations (yellow), negative affect (blue), and positive affect (red). Significant differences between pre- and post-lockdown measures are noted by brackets above the boxplots. Note: * *p* < .05, **, *p* < .01


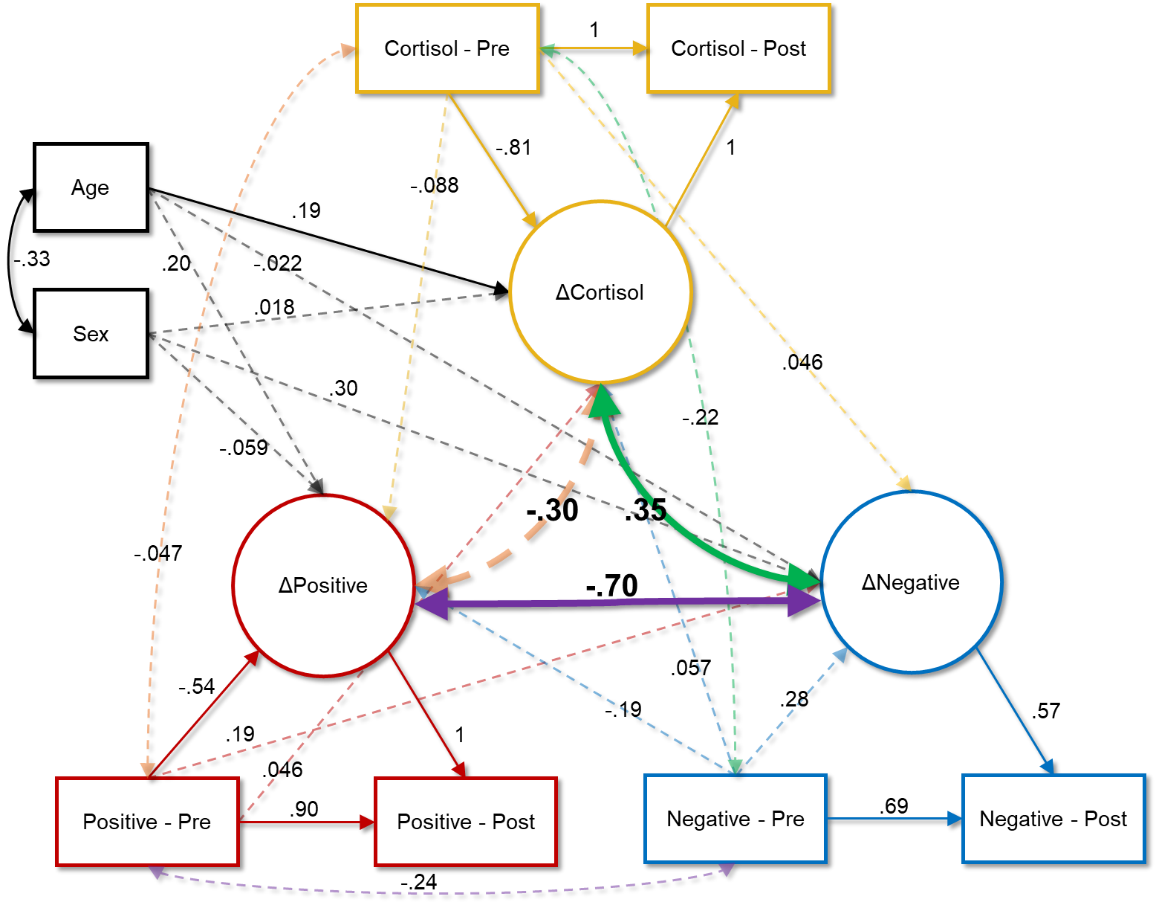


***Figure S2.*** **Correlational model of HCC and affect**

Results of the correlational model examining relationships between latent changes in hair cortisol concentrations, positive affect, and negative affect. Age and sex serve as control variables. Double-headed arrows show correlations, and single-headed arrows show directional predictive relationships. Dashed lines indicate non-statistically significant relationships, whereas solid lines show significant associations at the *p* < .05 level. All reported coefficients are standardized. Lines in **yellow** are indicative of cortisol-specific associations/estimates in which cortisol is predicting another variable; lines in **red** are indicative of positive affect-specific associations/estimates in which positive affect is predicting another variable; lines in **blue** are indicative of negative affect-specific associations/estimates in which negative affect is predicting another variable; secondary colors (**orange**, **purple** and **green**) indicate associations that are non-directional (i.e., correlations); **thicker lines** highlight the main parameters involved in hypothesis testing.


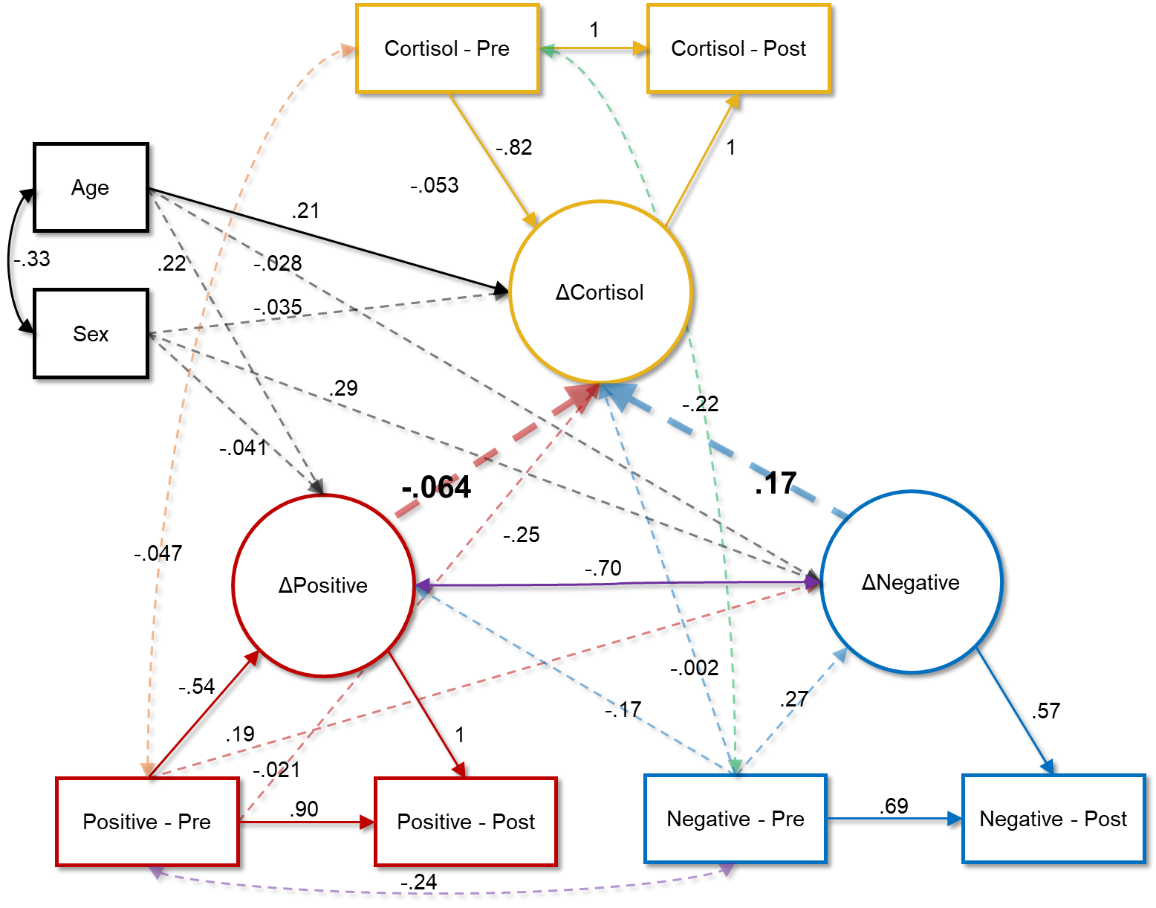


***Figure S3.*** **Predictive model wherein affect predicts cortisol**

Results of the model in which changes in positive and negative affect are predictors of changes in hair cortisol concentrations. Age and sex serve as control variables. Double-headed arrows show correlations, and single-headed arrows show directional predictive relationships. Dashed lines indicate non-statistically significant relationships, whereas solid lines show significant associations at the *p* < .05 level. All reported coefficients are standardized. Lines in **yellow** are indicative of cortisol-specific associations/estimates in which cortisol is predicting another variable; lines in **red** are indicative of positive affect-specific associations/estimates in which positive affect is predicting another variable; lines in **blue** are indicative of negative affect-specific associations/estimates in which negative affect is predicting another variable; secondary colors (**orange**, **purple** and **green**) indicate associations that are non-directional (i.e., correlations); **thicker lines** highlight the main parameters involved in hypothesis testing.


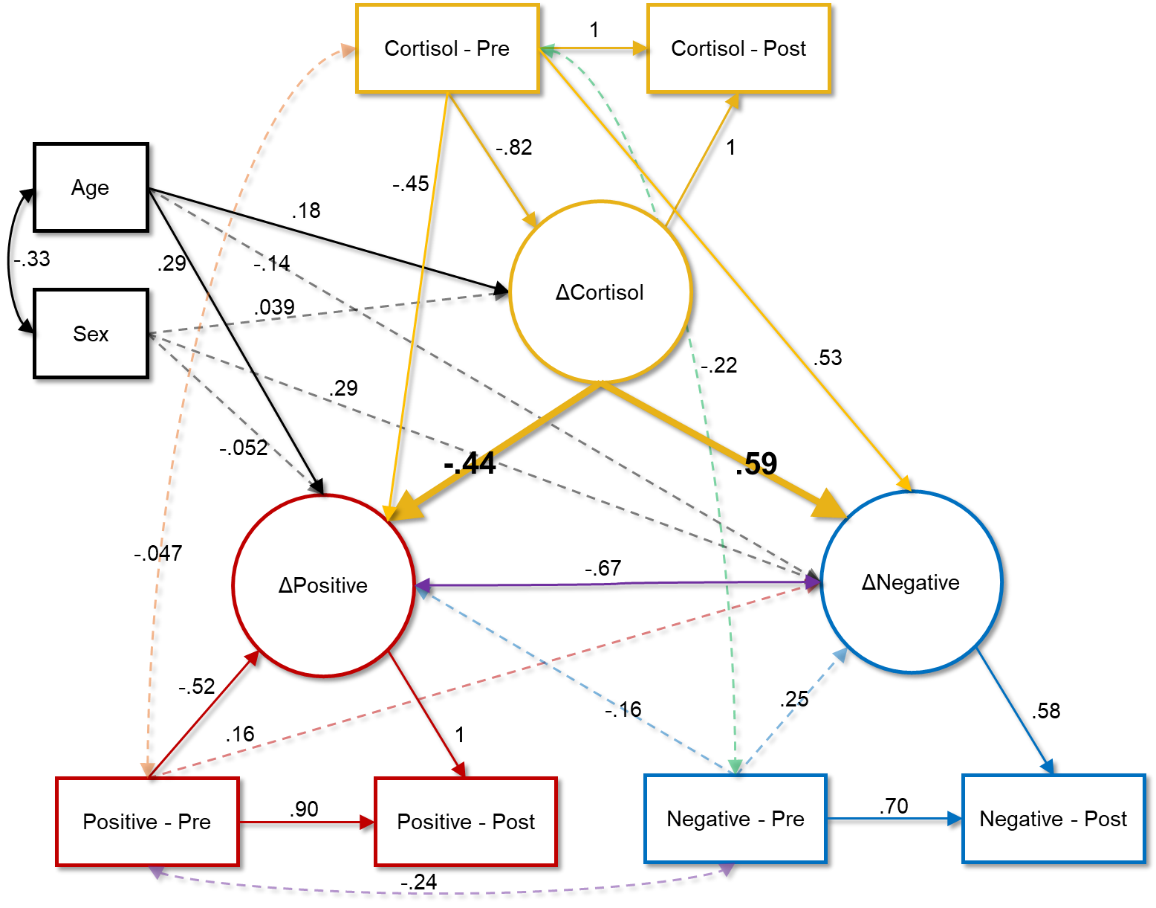


***Figure S4.*** **Predictive model wherein cortisol predicts affect**

Results of the model in which changes in hair cortisol concentrations predict changes in positive and negative affect. Age and sex serve as control variables. Double-headed arrows show correlations, and single-headed arrows show directional predictive relationships. Dashed lines indicate non-statistically significant relationships, whereas solid lines show significant associations at the *p* < .05 level. All reported coefficients are standardized. Lines in **yellow** are indicative of cortisol-specific associations/estimates in which cortisol is predicting another variable; lines in **red** are indicative of positive affect-specific associations/estimates in which positive affect is predicting another variable; lines in **blue** are indicative of negative affect-specific associations/estimates in which negative affect is predicting another variable; secondary colors (**orange**, **purple** and **green**) indicate associations that are non-directional (i.e., correlations); **thicker lines** highlight the main parameters involved in hypothesis testing.

**Table S1.** Demographic characteristics for the final sample included in analyses.

| **Characteristic** | | ***M*** | ***SD*** | | ***Range*** |
| --- | --- | --- | --- | --- | --- |
| **Age** (years) | | 14.70 | 2.04 | | 10.57 - 18.41 |
| **SES** (BSMSS total score) | | 51.37 | 9.82 | | 24.83 - 66.00 |
|  | |  |  | |  |
|  | | ***N*** | | ***%*** | |
| **Sex** | |  | |  | |
|  | Male | 13 | | 29.50 | |
|  | Female | 31 | | 70.50 | |
|  |  |  | |  | |
| **Race** | |  | |  | |
|  | White | 37 | | 84.10 | |
|  | Asian | 1 | | 2.30 | |
|  | Multiracial | 6 | | 13.60 | |
|  |  |  | |  | |
| **Ethnicity** | |  | |  | |
|  | Hispanic/Latino | 41 | | 93.20 | |
|  | Not Hispanic/Latino | 3 | | 6.80 | |
|  |  |  | |  | |
| **Mother’s Education** | |  | |  | |
|  | Partial High School | 1 | | 2.30 | |
|  | Partial College | 6 | | 13.60 | |
|  | College | 11 | | 25.00 | |
|  | Graduate | 26 | | 59.10 | |

**Table S2.** Hair type and hair treatment characteristics.

| **Hair Type** | ***N*** | **%** |
| --- | --- | --- |
| Straight | 22 | 50.00% |
| Wavy | 18 | 40.91% |
| Curly | 4 | 9.09% |
|  |  |  |
| **Hair Treatment** | ***N*** | **%** |
| Current hair dye | 9 | 20.45% |
| Current chemical treatment | 0 | 0% |
| Products (conditioner/gel/oil) | 25 | 56.82% |
|  |  |  |
|  | ***M* (*SD*)** | **Range** |
| Days since last wash | 1.57 (1.34) | 0 to 7 |
| Blow dry (days per week) | .46 (.78) | 0 to 4 |
| Straighten (days per week) | .60 (1.19) | 0 to 6 |

**Table S3.** Means, standard deviations, and ranges of values for hair cortisol concentrations and perceived positive and negative affect total scores pre- and post-lockdowns, as well as the difference between scores post- versus pre-lockdowns.

|  | **Pre-Lockdowns** | | | **Post-Lockdowns** | | | **Difference (post – pre)** | | |
| --- | --- | --- | --- | --- | --- | --- | --- | --- | --- |
|  | ***M*** | ***SD*** | **Range** | ***M*** | ***SD*** | **Range** | ***M*** | ***SD*** | **Range** |
| Hair Cortisol (pg/mL) | 4.92 | 4.03 | .75 – 19.65 | 6.43 | 2.28 | 2.85 – 14.10 | 1.51 | 3.88 | -14.10 – 7.65 |
| Positive Affect | 44.50 | 7.93 | 28 – 58 | 42.73 | 8.86 | 21 – 64 | -1.77 | 10.22 | -27 – 29 |
| Negative Affect | 27.07 | 8.06 | 15 – 45 | 29.89 | 11.72 | 15 – 59 | 2.82 | 6.71 | -12 – 19 |

**Table S4.** Correlation table showing associations between all variables of interest entered into the latent change score models.

|  | **1** | **2** | **3** | **4** | **5** | **6** | **7** | **8** |
| --- | --- | --- | --- | --- | --- | --- | --- | --- |
| **1. Pre-Cortisol** | — |  |  |  |  |  |  |  |
| **2. Pre-Positive** | -.05 | — |  |  |  |  |  |  |
| **3. Pre-Negative** | -.22 | -.24 | — |  |  |  |  |  |
| **4. Post-Cortisol** | .35^*^ | -.003 | -.06 | — |  |  |  |  |
| **5. Post-Positive** | -.06 | .26 | -.31^*^ | -.18 | — |  |  |  |
| **6. Post-Negative** | -.20 | -.02 | .83^***^ | .09 | -.56^***^ | — |  |  |
| **7. Age** | -.03 | -.16 | -.19 | .28 | .26 | -.25 | — |  |
| **8. Sex** | -.20 | .46^**^ | .10 | -.11 | -.02 | .30^*^ | -.33^*^ | — |

Note: sex was coded as 0 = male, 1 = female; *^*^ p < .05,* ^**^ *p* < .01, ^***^ *p* < .001

**Table S5.** Model fit indices for the three variants of the latent change score models: the correlational model, the model in which perceived changes in positive and negative affect predict changes in hair cortisol concentrations (PANAS 🡪 HCC), and the model in which changes in hair cortisol concentrations predict perceived changes in affect (HCC 🡪 PANAS).

|  | **Correlational Model** | **PANAS 🡪 HCC** | **HCC 🡪 PANAS** |
| --- | --- | --- | --- |
| **χ^2^**  ***(p)*** | — | .57  (*.75*) | .51  (*.77*) |
|  |  |  |  |
| **RMSEA**  **90% CI** | — | .00  [.00, .21] | .00  [.00, .20] |
|  |  |  |  |
| **CFI** | — | 1.00 | 1.00 |
|  |  |  |  |
| **SRMR** | — | .02 | .02 |
|  |  |  |  |
| **AIC** | 1897.94 | 1894.51 | 1894.45 |
| **ΔAIC** | — | 3.43 | 3.49 |
|  |  |  |  |
| **BIC** | 1976.44 | 1969.44 | 1969.38 |
| **ΔBIC** | — | 7.00 | 7.36 |
| ***R^2^* ΔCortisol** | .73^***^ | .76^***^ | .72^***^ |
| ***R^2^* ΔPositive** | .44^***^ | .44^***^ | .47^***^ |
| ***R^2^* ΔNegative** | .32^**^ | .31^**^ | .38^**^ |

^*^ *p* < .05; ^**^ *p* < .01; ^***^ *p* < .001 for *R*^2^ values

Note: The correlational model was saturated and thus did not have model fits for most statistics. Degrees of freedom for each of the reported chi square tests was 2; ΔAIC and ΔBIC metrics compare the specified directional predictive model to the correlational model; Only AIC and BIC fit indices are available for the correlational model because it was a saturated model structure.

“HCC” = hair cortisol concentration; “PANAS” = Positive Affect Negative Affect Schedule; “χ^2^” = chi square test of model fit; “(*p*)” = the *p*-value associated with the chi-square statistic; “RMSEA” = root mean square error of approximation; “90% CI” = the 90% confidence interval around the RMSEA (values in brackets are [lower bound, upper bound]); “SRMR” = standardized root mean square; “AIC” = Akaike information criterion; “BIC” = Bayesian information criterion

**Supplemental References**

Barratt, W. (2006). The Barratt simplified measure of social status (BSMSS): Measuring SES. *Unpublished Manuscript, Indiana State University*.

Contractor, A. A., Banducci, A. N., Dolan, M., Keegan, F., & Weiss, N. H. (2019). Relation of positive memory recall count and accessibility with post-trauma mental health. *Memory*, *27*(8), 1130–1143. https://doi.org/10.1080/09658211.2019.1628994

Frewen, P. A., Dean, J. A., & Lanius, R. A. (2012). Assessment of anhedonia in psychological trauma: Development of the Hedonic Deficit and Interference Scale. *European Journal of Psychotraumatology*, *3*(1), 8585. https://doi.org/10.3402/ejpt.v3i0.8585

Hu, L., & Bentler, P. M. (1999). Cutoff criteria for fit indexes in covariance structure analysis: Conventional criteria versus new alternatives. *Structural Equation Modeling: A Multidisciplinary Journal*, *6*(1), 1–55. https://doi.org/10.1080/10705519909540118

Hughes, A. A., & Kendall, P. C. (2009). Psychometric Properties of the Positive and Negative Affect Scale for Children (PANAS-C) in Children with Anxiety Disorders. *Child Psychiatry and Human Development*, *40*(3), 343–352. https://doi.org/10.1007/s10578-009-0130-4

Kievit, R. A., Brandmaier, A. M., Ziegler, G., van Harmelen, A.-L., de Mooij, S. M. M., Moutoussis, M., Goodyer, I. M., Bullmore, E., Jones, P. B., Fonagy, P., Lindenberger, U., & Dolan, R. J. (2018). Developmental cognitive neuroscience using latent change score models: A tutorial and applications. *Developmental Cognitive Neuroscience*, *33*, 99–117. https://doi.org/10.1016/j.dcn.2017.11.007

Ladouceur, C. D. (2020). *COVID-19 Adolescent Symptom & Psychological Experience Questionnaire*.

Landis, D., Courtney, W., Dieringer, C., Kelly, R., King, M., Miller, B., Wang, R., Wood, D., Turner, J. A., & Calhoun, V. D. (2016). COINS Data Exchange: An open platform for compiling, curating, and disseminating neuroimaging data. *NeuroImage*, *124*, 1084–1088. https://doi.org/10.1016/j.neuroimage.2015.05.049

Laurent, J., Catanzaro, S. J., Joiner, T. E. Jr., Rudolph, K. D., Potter, K. I., Lambert, S., Osborne, L., & Gathright, T. (1999). A measure of positive and negative affect for children: Scale development and preliminary validation. *Psychological Assessment*, *11*(3), 326–338. https://doi.org/10.1037/1040-3590.11.3.326

McNeish, D., & Matta, T. (2018). Differentiating between mixed-effects and latent-curve approaches to growth modeling. *Behavior Research Methods*, *50*(4), 1398–1414. https://doi.org/10.3758/s13428-017-0976-5

Monson, C. M., Price, J. L., Rodriguez, B. F., Ripley, M. P., & Warner, R. A. (2004). Emotional deficits in military-related PTSD: An investigation of content and process disturbances. *Journal of Traumatic Stress*, *17*(3), 275–279. https://doi.org/10.1023/B:JOTS.0000029271.58494.05

Muthen, L. K., & Muthén, B. O. (2012). *Mplus User’s Guide* (Seventh). Muthen & Muthen. https://www.statmodel.com/download/usersguide/Mplus%20user%20guide%20Ver_7_r6_web.pdf

Ouellet-Morin, I., Laurin, M., Robitaille, M.-P., Brendgen, M., Lupien, S. J., Boivin, M., & Vitaro, F. (2016). Validation of an adapted procedure to collect hair for cortisol determination in adolescents. *Psychoneuroendocrinology*, *70*, 58–62. https://doi.org/10.1016/j.psyneuen.2016.05.002

Ramirez, J., Elmofty, M., Castillo, E., DeRouen, M., Shariff-Marco, S., Allen, L., Gomez, S. L., Nápoles, A. M., & Márquez-Magaña, L. (2017). Evaluation of cortisol and telomere length measurements in ethnically diverse women with breast cancer using culturally sensitive methods. *Journal of Community Genetics*, *8*(2), 75–86. https://doi.org/10.1007/s12687-016-0288-y

Raudales, A. M., Short, N. A., & Schmidt, N. B. (2019). Emotion dysregulation mediates the relationship between trauma type and PTSD symptoms in a diverse trauma-exposed clinical sample. *Personality and Individual Differences*, *139*, 28–33. https://doi.org/10.1016/j.paid.2018.10.033

Scott, A., Courtney, W., Wood, D., De la Garza, R., Lane, S., Wang, R., King, M., Roberts, J., Turner, J. A., & Calhoun, V. D. (2011). COINS: An Innovative Informatics and Neuroimaging Tool Suite Built for Large Heterogeneous Datasets. *Frontiers in Neuroinformatics*, *5*. https://doi.org/10.3389/fninf.2011.00033

Seligowski, A. V., & Orcutt, H. K. (2016). Support for the 7-factor hybrid model of PTSD in a community sample. *Psychological Trauma: Theory, Research, Practice, and Policy*, *8*(2), 218–221. https://doi.org/10.1037/tra0000104

Short, S. J., Stalder, T., Marceau, K., Entringer, S., Moog, N. K., Shirtcliff, E. A., Wadhwa, P. D., & Buss, C. (2016). Correspondence between hair cortisol concentrations and 30-day integrated daily salivary and weekly urinary cortisol measures. *Psychoneuroendocrinology*, *71*, 12–18. https://doi.org/10.1016/j.psyneuen.2016.05.007

Tworoger, S. S., & Hankinson, S. E. (2006). Use of biomarkers in epidemiologic studies: Minimizing the influence of measurement error in the study design and analysis. *Cancer Causes & Control*, *17*(7), 889–899. https://doi.org/10.1007/s10552-006-0035-5

Wang, W., Moody, S. N., Kiesner, J., Tonon Appiani, A., Robertson, O. C., & Shirtcliff, E. A. (2019). Assay validation of hair androgens across the menstrual cycle. *Psychoneuroendocrinology*, *101*, 175–181. https://doi.org/10.1016/j.psyneuen.2018.10.029

Wang, W., Moody, S. N., & Shirtcliff, E. A. (2016). Noninvasive hair assay for sex hormones: Preliminary protocol validation. *Psychoneuroendocrinology*, *71*, 45. https://doi.org/10.1016/j.psyneuen.2016.07.119

Wennig, R. (2000). Potential problems with the interpretation of hair analysis results. *Forensic Science International*, *107*(1–3), 5–12. https://doi.org/10.1016/S0379-0738(99)00146-2
